# Development and validation of the S-TIMHSS: a quality metric to inform and evaluate interventions to (re)build trust

**DOI:** 10.3389/fpubh.2025.1568836

**Published:** 2025-04-30

**Authors:** Samantha B. Meyer, Jerrica Little, Paul R. Ward, Patrick Brown, Michael Calnan

**Affiliations:** ^1^School of Public Health Sciences, University of Waterloo, Waterloo, ON, Canada; ^2^Centre for Public Health, Equity and Human Flourishing, Torrens University, Adelaide, SA, Australia; ^3^Department of Sociology, University of Amsterdam, Amsterdam, Netherlands; ^4^School of Social Policy, Sociology and Social Research, University of Kent, Canterbury, United Kingdom

**Keywords:** healthcare, policy, doctor, trust, measurement, intervention, evaluation

## Abstract

**Introduction:**

Public acceptance of health messaging, recommendations, and policy is heavily dependent on the public’s trust in doctors, health systems and health policy. Any erosion of public trust in these domains is thus a concern for public health as it can no longer be assumed that the public will follow official health recommendations. In response, the health policy and health services communities have emphasized a commitment to (re)building trust in healthcare. As such, measures of trust that can be used to develop and evaluate interventions to (re)build trust are highly valuable. In 2024, the Trust in Multidimensional Health System Scale (TIMHSS) was published, providing the first measure of trust in healthcare that includes doctors, the system and health policy within a single measure. This measure can effectively facilitate research on trust across diverse populations. However, it is limited in its application because results cannot be directly added together for a total trust score. Further, at 38-items, it is burdensome for respondents and analysts, particularly when being used as a repeat measure in an applied setting. The aim of the present work was to develop a shortened measure of trust in healthcare for use in applied settings.

**Methods:**

Survey data were collected (*N* = 512; in Sept 2024) to reduce the number of items and to test if the factor structure was consistent with the original TIMHSS. Several statistical criteria were used to support item reduction (i.e., correlated errors, measurement invariance, inter-item correlations, factor loadings and communalities, item-total correlation, and skewness), as well as an exercise testing the content validity ratio (CVR). We then tested a three-factor model based on the 18 items that remained following the CVR and statistical test metrices to finalize the measure.

**Results:**

The S-TIMHSS is an 18-item scale that allows for direct scoring of trust items for applied research. It preserves the content, convergent, and criterion validity of the original 38-item version.

**Discussion:**

We recommend the measure be used by health policy makers and practitioners as a quality metric to inform and evaluate interventions which aim to (re)build trust in doctors, health systems and health policy.

## 1 Introduction

Patients’ and publics’ acceptance of health information is heavily dependent on their perceptions of the trustworthiness of healthcare providers, health systems and health policy [e.g., see Majid, Wasim (1)]. Growing evidence regarding public criticism of democratic socio-political systems, including healthcare, is thus a cause of concern from a population health perspective. These criticisms have been attributed to false or misleading information in digital and physical environments (2), changes in what the public consider to be ‘legitimate’ information in the context of social media (3), and the public placing trust in sources of alternative expertise that run counter to that of information provided by credentialed healthcare providers. These more contemporary determinants of trust build on longstanding factors associated with declining trust in healthcare [see (4)], well publicized instances of medical misconduct [e.g., see Searle and Rice (5)], and systemic factors (e.g., racism, discrimination) that have led some of the public to question their trustworthiness [e.g., (6)].

Trust is critical for population health. A 2017 systematic review examining if patients’ trust in providers is linked to clinical outcomes reported that trust is associated with beneficial health behaviors (medication adherence, screening behavior, health promoting lifestyle, online search behavior), fewer symptoms, and higher quality of life (7). Building trust in health systems has also been suggested as a mechanism for eliminating health disparities (8) and has implications for health system costs if one considers that a lack of trust leads to requests for second opinions or alternatively, disengagement with care leading to increased morbidity. Strategies to build patient trust should thus be a priority for democratic countries where low trust is negatively influencing population health, and particularly among disadvantaged communities [e.g., see Ike, Burns (9)]. To respond to challenges of trust, we need data to inform tailored strategies that are context and population specific.

As a fundamental dimension of the effectiveness of a health system, methods to understand and respond to challenges of trust in health settings warrant serious consideration in clinical practice. To date, existing tools used to measure trust in a clinical setting have limitations in their ability to inform change at the doctor, system, *and* policy level through a single measure (10), or they are too long and thus burdensome for respondents and analysts (11). The aim of the present paper is to report the development and validation of a shortened measure of trust in healthcare that includes doctors, the health system, and health policy that can be used in a clinical setting for ongoing monitoring and evaluation. Working with the existing 38-item Trust in Multi-dimensional Healthcare Systems Scale (11), we explore whether it is possible to reduce items while preserving the content, convergent and criterion validity. In doing so, we provide a measure of trust for use in clinical settings that can inform and be used to evaluate evidence-based responses to (re)building trust that are context and population specific.

### 1.1 Conceptual framework

Conceptual framework. Trust is a complex multidimensional concept consisting of both a rational component (arising from experience) and a non-rational component based on intuition and emotion (12). Despite agreement that trust in the context of healthcare is multidimensional, the dimensions of trust in existing measures vary (10). Within the present work, we identify trust as contingent on two critical dimensions – competence and shared interests (13, 14). That is, we consider patient/public trust to be largely based on whether the individuals or institutions for whom they are being called to trust are competent and will act in the best interest of the patient/public. The present work also recognizes that trust occurs at two distinct levels – institutional and interpersonal trust (15). That is, in the context of healthcare, trust extends beyond relationships between patients and providers to include health systems and the macro-level structures that govern their practice.

Conceptualizing and thus researching trust in the context of health is challenging. The conditions under which one makes a decision to trust are so varied given the spectrum of services that fall under the umbrella of health. For example, the decision to accept a new vaccine is very different than the decision to change blood pressure medications. In both cases, patients are being asked to trust, but the perceived risks and benefits, and factors influencing trust, will vary. As such, strategies for fostering trust and increasing the acceptance of health messaging need to be driven by, and responsive to, the unique context and populations of focus. Within the present work we define trust as *“A’s expectation that a trustee B will display behavior X in situation/context Y”* (16) *(p. 2), acknowledging the importance of considering* the context under which one is being asked to trust in the measurement of the construct. The original 38-item TIMHSS can be used to identify the role of trust in patient decisions as it varies by context – e.g., the acceptance of preventative measures or engagement with clinical services – or to investigate associations with health outcomes. However, it needs to be shortened for practical use in clinical settings.

## 2 Materials and methods

### 2.1 Background to Trust in Multi-dimensional Healthcare Systems Scale

The Trust in Multidimensional Healthcare System Scale (11) is a global measure of trust in healthcare and can be used to measure trust over time at a population level, or used within specific subpopulations, to inform interventions to (re)build trust. It was designed for use among staff and providers in clinical healthcare settings to support and extend the measurement of patient experience, and thus the measure we chose to shorten for use in clinical settings. Currently a 38-item scale, the TIMHSS is the first measure of trust in healthcare that looks at doctors, the system and health policy within a single measure. Analyses demonstrate support for the validity of the measure in that it predicts patient acceptance of medications or treatment plans, disclosure of medically relevant information, new vaccine acceptance, and not delaying access to care or seeking a second opinion. However, while the current measure covers several elements of trust in healthcare and can effectively facilitate research on trust across different populations, the factor structure includes covarying residual terms, meaning that results from the survey cannot be directly scored for a total trust point, nor can statistical means be compared across sub-groups. Modeling covarying error terms is feasible for research purposes and permits a fuller understanding of the trust construct. However, stakeholders interested in deriving a trust score and using it as a quality metric require a less complex structure that can be easily summarized and does not require hundreds of participants to model. Further, 38-items to measure a single construct is too burdensome for respondents and will limit response rates and survey completion. The present work was conducted to develop a shorter survey with a subset of items to reduce the time burden for respondents and analysts, and to allow for direct scoring of trust items for applied research.

### 2.2 Statistical analysis

#### 2.2.1 Item content validity ratio

For each of the 38 items in the original TIMHSS, N = 4 authors with expertise in the field of trust in health systems provided an independent rating of content relevance using the following scale: 1 = not necessary, 2 = useful, but not necessary and, 3 = essential. Using the number of experts who scored the item as ‘3’ for the reference point (ne), the CVR for each item was calculated as (ne – N/2)/(N/2) (17). Items with a CVR value of 0 or lower were considered for deletion from the scale, as this meant that two or fewer authors rated it as a ‘3’. Conversely, items with a CVR of 1 were retained, as all four authors believed the item to be essential.

#### 2.2.2 Statistical tests for item reduction

Given that the purpose of the shortened scale was to increase the feasibility of the tool in practice, a series of statistical tests were then reviewed with this goal in mind:

##### 2.2.2.1 Correlated errors

Variables requiring shared error terms were removed from the scale because these terms added substantial complexity to the model, making it difficult to replicate across studies (18). Shared error terms also prevented items from being added together to form a total score, since additive scales assume items have independent random error. These issues made the scale difficult for organizations to use in practice, since straightforward, easy-to-interpret comparisons across different samples could not be made. In the 38-item scale (11), correlated error terms needed to be specified for items within the same theoretical dimension, e.g., items in ‘question 14’ (patient focus of providers). By reducing the number of variables in each dimension, shared error terms were no longer required to produce adequate model fit.

##### 2.2.2.2 Measurement invariance

Items that varied in their measurement properties across demographic subpopulations were considered for removal from the scale, since these items were not directly comparable across groups (19). Two types of measurement invariance were reviewed for item reduction: metric and scalar invariance. Metric invariance assesses whether item loadings are equivalent between groups (19), while scalar invariance tests if intercepts (item means) are equal between groups. Items demonstrating metric variance in relation to gender identity and sexual orientation from the derivation paper (11) were considered for removal, followed by items with scalar variance.

##### 2.2.2.3 Inter-item correlations

Between items that were highly correlated in a subscale (>0.80), only one or two were retained (20). For the purposes of shortening the scale, the inter-item correlation threshold was lowered to 0.70, which is still considered to be a strong correlation coefficient (21, 22).

##### 2.2.2.4 Item communalities

In social science research, communalities among items in a scale tend to range from 0.40–0.70, and so a minimum item communality of 0.40 is recommended (22, 23). A more stringent cut-off value for communalities is 0.60 (24) and so to shorten the scale, items with communalities below this value were considered for removal.

Values for these statistics were obtained from the second sample described in Meyer, Brown (11).

#### 2.2.3 Confirmatory factor analysis

Before proceeding with CFA, the suitability of the dataset for item reduction was evaluated through the Keyer-Maiser-Olkin (KMO) Mean Square Approximation (MSA) test and Bartlett’s test of sphericity. Multivariate normality was assessed using the Mardia skewness and kurtosis tests; if the tests revealed significant non-normality, the maximum likelihood estimation with robust standard errors and a Satorra-Bentler scaled test statistic (MLM) was used to derive CFA models.

Reliability of the scale was assessed using McDonald’s hierarchical (ω_h_) and total (ω_t_) omega coefficients for a three-factor structural equation model (SEM), which was derived from an exploratory model with Schmid Leiman general factor loadings, generated using the ‘omega’ function in R (default settings applied). As a rule, both omega coefficients should ideally be greater than 0.80 (25). Reliability at the item-level was evaluated through average inter-item correlations (IIC), item-total correlations (ITC) with the item dropped, and standardized Cronbach’s alpha coefficient (*α*), which were calculated separately for items within each scale (doctor, system, and policy). Cronbach’s alpha coefficients of at least 0.80 and IIC & ITC of at least 0.30 are recommended (24, 26).

Based on the original 38-item scale (11), we expected a correlated three-factor structure consisting of doctor, system, and policy dimensions to be the best fit for the data. To establish whether model performance was indeed the best for three-factors, a series of alternative solutions were tested, including an uncorrelated three-factors model, as well as nested one-and two-factor models (models nested by setting the ‘doctor’ and ‘system’ factor parameters to be the same and all three factor parameters to be the same, respectively). Model fit criteria were selected based on recommended values of >0.95 on the Comparative Fit Index (CFI), >0.90 on the Tucker-Lewis Index (TLI) ≤ 0.08 on the Root Mean Square Error of Approximation (RMSEA), and <0.08 on the Standardized Root Mean Squared Residual (SRMR) (27, 28). To compare model fit directly, the Akaike information criterion (AIC) and Bayesian information criterion (BIC) were evaluated, with lower values indicating better model fit.

#### 2.2.4 Validity testing

To ensure that the shortened version of the TIMHSS produced the same patterns of association as the original scale, the tests used to establish validity in the original paper (11) were repeated in this study.

##### 2.2.4.1 Convergent validity

Spearman’s rank correlation tests were calculated separately for each of the three TIMHSS factors and the following variables: Two questions measuring satisfaction were included in the survey to determine convergent validity: (a) “I am perfectly satisfied with the health care I have been receiving (29)” from 1 (strongly agree) to 6 (strongly disagree), (b) “There are some things about the health care I have been receiving that could be better” from 1 (strongly agree) to 6 (strongly disagree) and, (c) the Trust in Physician Scale (40) from 10 to 43 (higher scores represent lower trust in physicians).

##### 2.2.4.2 Discriminant validity

Point biserial correlations were calculated between each of the three TIMHSS factors and the following two questions: “I never question the medical advice I am given by my doctor” (agree/disagree) and “I have no choice but to follow the recommendations provided by my doctor” (agree/disagree).

##### 2.2.4.3 Criterion validity

Logistic regression models were conducted for the following five dependent variables: (a) “I always follow doctors’ recommendations,” (b) “I would be willing to accept a new vaccine if my doctor recommended it,” (c) “During the past 12 months, was there any time you chose not to get the medical care you needed?,” (d) “I always tell my doctor the truth when they ask for information relevant to my healthcare” and, (e) “Have you changed physicians in the past or sought a second opinion due to concerns about care?” For each of the regression models, “yes” or “agree” was the reference group for the dependent variable and the doctor, system, and policy factors were entered as separate independent variables.

#### 2.2.5 Measurement invariance

To determine whether the 18-item TIMHSS was invariant between women and non-women, model fit was compared at the scalar and metric levels.

All statistics were performed in R version 4.3.1 (30). The list of R packages used to perform statistical analyses include the following: corrplot (31), psych (32), lavaan (33), MVN (34), semTools (35), and tidyverse (36).

## 3 Results

### 3.1 Item reduction

Item-level properties used to determine variable reduction for each of the 38 TIMHSS questions are summarized below in Table 1.

**Table 1:** Item-level properties for 38 TIMHSS questions and the decision to keep or remove the question for the shortened scale.

Factor	TIMHSS Item	CVR	Correlated error terms (Yes)	Metric variance (Yes)	Scalar variance (Yes)	Inter-item correlation >0.70	Communality	Decision
2 (doctor)	12a	1	–	–	Y	–	0.50	Keep
	12b	–1	–	–	–	12c	0.67	Remove
	12c	−0.5	–	–	–	12b	0.64	Keep
	12d	−0.5	–	–	–	12f	0.65	Keep
	12e	1	–	Y	Y	–	0.57	Keep
	12f	−0.5	–	Y	Y	12rd, 12rg, 12rh, 12rj, 12rl, 12rm	0.72	Remove
	12 g	−1	–	–	–	12f, 12h, 12i, 12j	0.72	Remove
	12 h	0	–	–	–	12f, 12g, 12i, 12j, 12L	0.74	Remove
	12i	0	–	–	–	12g, 12h	0.66	Keep
	12j	−1	–	–	–	12f, 12g, 12h, 12L, 12m	0.73	Remove
	12 k	0	–	–	–	–	0.60	Keep
	12 L	−1	–	–	–	12f, 12h, 12j, 12m	0.72	Remove
	12 m	−0.5	–	–	–	12f, 12j, 12L	0.66	Keep
3 (policy)	13a	−0.5	–	–	–	–	0.50	Keep
	13d	0	–	–	–	13e, 13f	0.76	Keep
	13e	0.5	–	–	–	13d	0.63	Keep
	13f	−1	–	–	–	13d	0.67	Remove
1 (system)	14b	−0.5	Y	–	Y	–	0.51	Remove
	14c	0	Y	–	–	–	0.56	Keep
	14d	−1	Y	–	Y	–	0.54	Remove
	14e	−1	Y	–	Y	–	0.54	Remove
	14f	−1	Y	–	–	–	0.60	Keep
	15a	0	Y	Y	Y	15c, 15d, 15g	0.59	Remove
	15c	0	Y	–	–	15a, 15d, 15g	0.63	Remove
	15d	−1	Y	–	Y	15a, 15c, 15g, 15h	0.63	Remove
	15e	0.5	–	–	Y	–	0.51	Keep
	15f	−0.5	–	Y	Y	15g, 15h	0.59	Remove
	15 g	1	–	–	–	15a, 15c, 15d, 15f, 15h	0.71	Keep
	15 h	0.5	–	–	–	15d, 15f, 15g	0.66	Keep
	16a	0.5	Y	–	Y	16b, 16c	0.59	Keep
	16b	0.5	Y	–	–	16a, 16c, 16d, 16f	0.60	Remove
	16c	0.5	Y	Y	Y	16a, 16b, 16d, 16f	0.63	Remove
	16d	0.5	Y	–	–	16b, 16c, 16e, 16f	0.65	Keep
	16e	0	Y	Y	Y	16d, 16f	0.64	Remove
	16f	−1		–	–	16b, 16c, 16d, 16e	0.64	Keep
	17a	−1	Y	–	Y	–	0.47	Remove
	17b	−0.5	Y	–	–	–	0.54	Remove
	17c	−1	–	–	–	–	0.46	Remove

Correlated errors, measurement variance, inter-item correlations, and communalities were derived from sample 2 of the original scale development study (11).

Three items (12a, 12e, 15 g) had a CVR = 1, meaning that all four authors believed it to be essential to the scale, and so these items were retained automatically. The decision to keep or remove the remaining questions was made based on a combination of item properties. For sets of items sharing correlated error terms, as well those with IICs of 0.70 or greater, only one or two were kept. Items with measurement invariance were preferred, followed by those with CVRs above 0 and communalities above 0.60. For the policy factor, only one item could be removed, as three are needed to enable factor identification; in this case, the worst-performing item was removed (13d). Following this process, 18 questions were retained for the shortened scale.

### 3.2 Descriptive statistics

Sociodemographic characteristics are reported below in Table 2. While a fairly symmetric distribution was observed for age and income groups, the sample was skewed more toward individuals identifying as white (65%), heterosexual (84%), and a woman (50%) or man (50%).

**Table 2:** Sociodemographic characteristics of study sample (*N* = 512).

Variable	Response option	% of sample (*n*)
Gender identity	Man	49.8 (255)
	Woman	49.6 (254)
	Agender; Genderqueer/Gender non-conforming/Gender non-binary; Transgender; Queer	2.2 (11)
Sexual orientation	Heterosexual	84.0 (430)
	Gay man	4.5 (23)
	Lesbian	1.9 (10)
	Bisexual/Pansexual	3.5 (18)
	Asexual/Aromantic	1.4 (7)
	Questioning; Another sexual orientation not listed	1.4 (7)
	Prefer not to answer	3.3 (17)
Ethnicity	Caucasian	334 (65.2)
	Asian	17.6 (90)
	First Nation, Inuit, Metis	1.6 (8)
	Black/African Canadian	12.3 (63)
	South/Central American	1.2 (6)
	Arab	0.9 (4)
	Another ethnicity not listed	3.7 (19)
Age group	18–24	10.7 (55)
	25–34	16.2 (83)
	35–44	16.6 (85)
	45–54	20.5 (105)
	55–64	17.4 (89)
	65–74	13.9 (71)
	75+	4.7 (24)
Gross household income	<$19,999	6.3 (32)
	$20,000–$39,999	12.5 (64)
	$40,000–$59,999	14.3 (73)
	$60,000–$79,999	15.0 (77)
	$80,000–$99,999	12.7 (65)
	$100,000–$119,999	9.8 (50)
	$120,000–$139,999	5.9 (30)
	$140,000–$159,999	3.7 (19)
	>$160,000	10.6 (54)
	Prefer not to answer	9.4 (48)

Multiple options could be selected for gender identity and so percentage exceeds 100.

Item means, standard deviations (SDs), skew, and kurtosis are summarized below in Table 3.

**Table 3:** Means, standard deviations, skew, and kurtosis values for 18 TIMHSS items (*N* = 512).

TIMHSS item	Mean	SD	Skew	Kurtosis
12a	2.33	1.01	0.57	−0.14
12c	2.53	1.09	0.42	−0.58
12d	2.38	1.04	0.59	−0.29
12e	2.21	0.98	0.63	0.05
12i	2.78	1.14	0.24	−0.85
12 k	2.38	0.97	0.55	−0.18
12 m	2.28	1.00	0.59	−0.09
13a	3.60	1.31	−0.58	−0.87
13d	3.57	1.28	−0.57	−0.82
13e	3.49	1.30	−0.52	−0.88
14c	2.09	0.98	0.93	0.62
14f	2.06	0.97	1.04	1.00
15e	2.08	1.03	0.90	0.28
15 g	2.63	1.13	0.39	−0.73
15 h	2.34	0.98	0.84	0.45
16a	2.33	1.00	0.72	0.12
16d	2.39	1.05	0.61	−0.39
16f	2.38	0.99	0.68	0.24

Range of scores is between 1 and 5, with ‘1’ indicating the most trust and ‘5’ the least trust.

**Figure 1:**
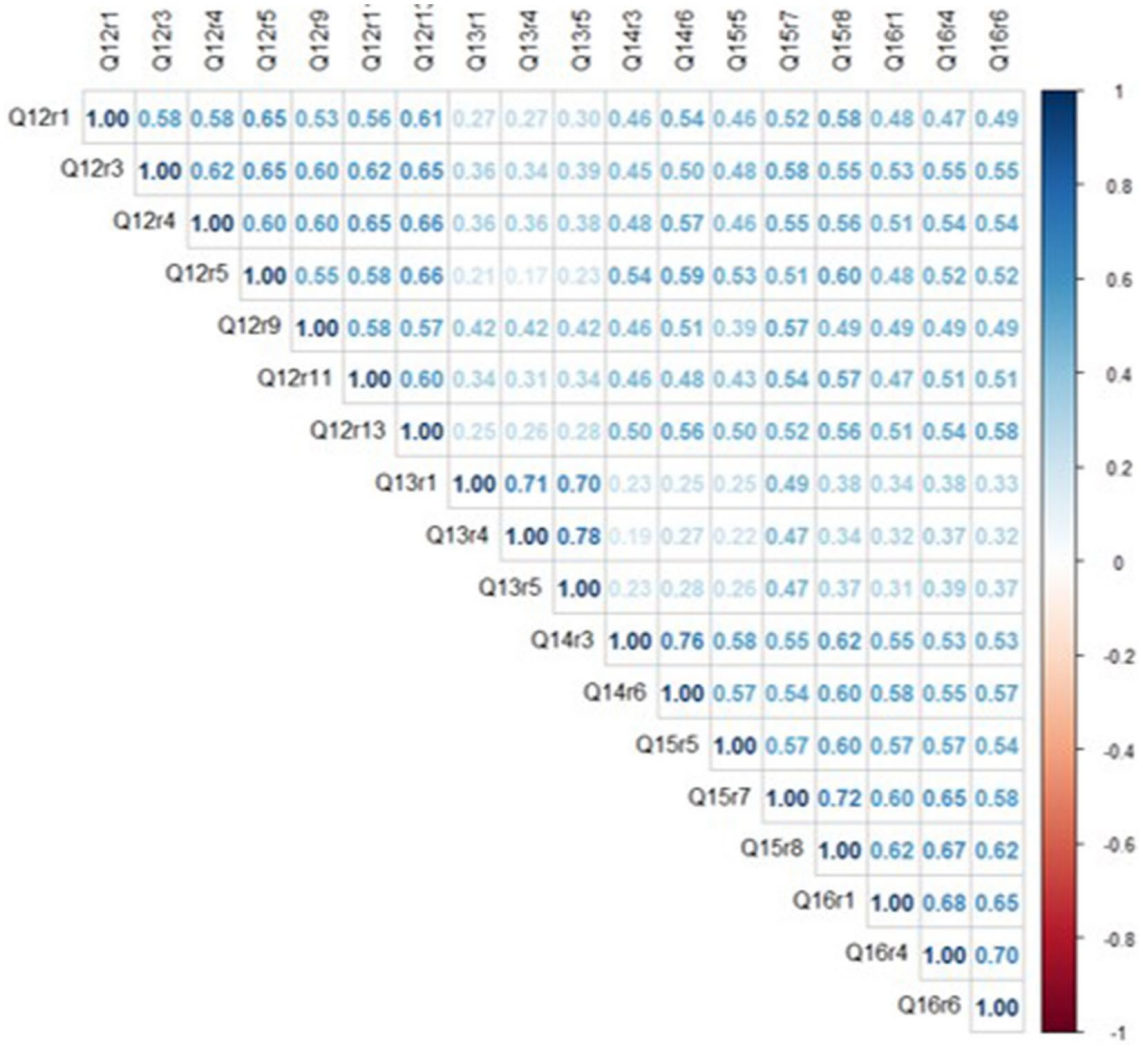
Spearman correlation matrix plot of 18 TIMHSS items.

On average, responses to survey questions clustered around the middle of the distribution, as mean scores for items ranged between 2.1–3.6, with skewness values below ‘1’ for all but one variable. There was also useful variance in the response distributions, as all items had SDs around ‘1’ and kurtosis values were below ‘1’ for all but one variable. Overall, average trust scores were highest for items belonging to the doctor (questions 12) and system (questions 14, 15, and 16) factors and lowest for those in the policy factor (question 13). The correlation matrix is shown below in Figure 1.

Inter-item correlations (IIC) ranged from *ρ* = 0.17–0.78, with an average IIC of ρ = 0.49. Among six item pairs with strong correlations (ρ ≥ 0.70), each pair was clustered within the same conceptual category (e.g., questions 14c & 14f), consistent with the theoretical organization of trust domains (11). Overall, because moderate associations were detected for most item pairs, it is apparent that the 18 TIMHSS variables share a common underlying ‘trust’ trait without being overly redundant with each other.

### 3.3 Reliability

Based on a three-factor SEM, the internal consistency reliability of a general second-order factor was ω_h_ = 0.88, and after adding the factor-specific variance of the doctor, system and policy domains, the total omega was ω_t_ = 0.96. Altogether, these coefficients suggest that the 18-item TIMHSS is a consistent representation of underlying trust in healthcare systems.

All items had standardized alpha coefficients greater than 0.80, as well as IIC and ITC values greater than 0.30, meaning that the scale would not benefit from removing any items (Table 4).

**Table 4:** Item-level reliability indices for 18 TIMHSS items (*N* = 512).

TIMHSS Item	Average ITC	ITC without item	Standardized α
12a	0.62	0.71	0.91
12c	0.60	0.77	0.90
12d	0.60	0.77	0.90
12e	0.61	0.75	0.90
12i	0.62	0.71	0.91
12 k	0.61	0.73	0.91
12 m	0.60	0.77	0.90
13a	0.79	0.74	0.88
13d	0.70	0.81	0.83
13e	0.70	0.81	0.83
14c	0.61	0.71	0.92
14f	0.61	0.72	0.92
15e	0.61	0.70	0.92
15g	0.60	0.75	0.91
15h	0.59	0.80	0.91
16a	0.60	0.75	0.91
16d	0.60	0.77	0.91
16f	0.61	0.73	0.92

All values are calculated for items within each scale.

### 3.4 Confirmatory factor analysis

**Figure 2:**
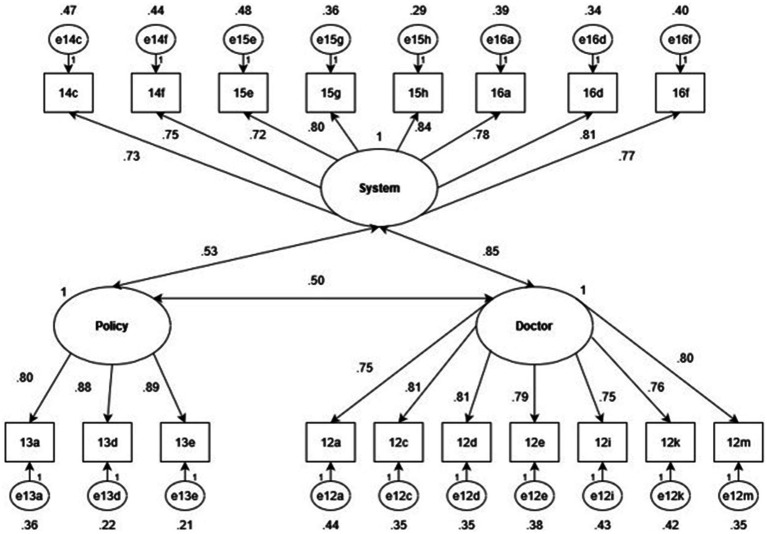
Standardized factor and error loadings of a three-factor CFA with doctor, system and policy latent factors.

The KMO MSA = 0.95, demonstrating the suitability of the dataset for item reduction. Mardia’s skewness and kurtosis (*p* = 0.0) tests were both statistically significant, indicating that the dataset was not multivariate normal. To correct for a non-normal distribution, the MLM estimation method was specified for CFA models. The three-factor CFA is shown below in Figure 2.

Item variances of all 18 TIMHSS items were explained strongly by the underlying factor structure, as all factor loadings were greater than 0.70. Moderate correlations were calculated between the policy factor and the doctor (*r* = 0.50) and system (*r* = 0.43) factors, as well as a strong correlation between the doctor and system factors (*r* = 0.85). While such a strong correlation may suggest that the two factors may be better modeled as one, the two-factor structure was inferior to the three-factor model, as indicated by below in Table 5.

**Table 5:** Robust model fit statistics for alternative factor structures of the 18-item TIMHSS.

Model	DF	CFI	TLI	RMSEA	SRMR	AIC	BIC
Correlated three-factor	132	0.949	0.941	0.070	0.044	21186.85	21352.14
Uncorrelated three-factor	135	0.852	0.832	0.118	0.346	21806.54	21959.12
Correlated two-factor (doctor + system, policy)	134	0.897	0.883	0.098	0.057	21521.79	21678.61
Unidimensional	135	0.794	0.765	0.139	0.086	22188.19	22340.77

The robust model fit statistics are displayed for CFI, TLI, and RMSEA.

The correlated three-factor model demonstrated excellent model fit (CFI = 0.95, TLI > 0.90, RMSEA<0.08, and SRMR<0.08) and was superior to all alternative solutions, confirming that it is the best structure to explain patterns in the observed dataset.

### 3.5 Validity tests

#### 3.5.1 Convergent validity

Spearman’s rank correlation coefficients for “I am perfectly satisfied with the health care that I have been receiving” were *r_s_* = 0.56 (*p* < 0.0001) for the Doctor factor, *r_s_* = 0.61 (*p* < 0.0001) for the System factor, and *r_s_* = 0.33 (*p* < 0.0001) for the Policy factor. These estimates are almost identical to those of the 38-item TIMHSS (11). Regarding the question, “There are some things about the health care I have been receiving that could be better,” Spearman’s rank correlation coefficients were *r_s_* = −0.25 (*p* < 0.0001) for the Doctor factor, *r_s_* = −0.23 (*p* < 0.0001) for the System factor, and *r_s_* = −0.19 (*p* < 0.0001) for the Policy factor. While these estimates are lower than those reported in the original scale development paper (11), in both cases, the associations were weak. Finally, the correlation coefficients between the Doctor, System, and Policy factors and the Trust in Physician scale were as follows: *r_s_* = 0.65 (*p* < 0.0001), *r_s_* = 0.71 (*p* < 0.0001), and *r_s_* = 0.38 (*p* < 0.0001). Unlike the previous question, compared to the original study (11) the estimates in this study were noticeably greater. For instance, the correlation between the Trust in Physicians scale and the System factor was moderate in the previous study (*r_s_* = 0.53) but strong in this one.

#### 3.5.2 Discriminant validity

For the Doctor, System and Policy factors, the point biserial correlation coefficients were as follows for the question “I never question the medical advice I am given by my doctor”: *r* = 0.34 (*p* < 0.0001), *r* = 0.33 (*p* < 0.0001), and *r* = 0.25 (*p* < 0.0001), respectively. These results are consistent with the pattern observed in the original study (11). Similarly, for the question, “I have no choice but to follow the recommendations provided by my doctor,” the correlation coefficients were non-existent or very weak: associations with the Doctor [*r* = 0.06 (*p* = 0.15)] and System [*r* = 0.06 (*p* = 0.20)] factors were statistically insignificant, but statistically significant for the Policy factor [*r* = 0.18, (*p* < 0.0001)].

#### 3.5.3 Criterion validity

Results from logistic regressions predicting dependent variables for criterion validity are summarized below in Table 6.

**Table 6:** Slope coefficients, standard errors, and statistical significance of the doctor, system, and policy factors in predicting dependent variables selected for criterion validity.

Question	Doctor β (SE)	System β (SE)	Policy β (SE)
I always follow doctors’ recommendations (disagree vs. agree)	0.10 (0.03)***	0.10 (0.03)***	0.05 (0.04)
I would be willing to accept a new vaccine if my doctor recommended it (disagree vs. agree)	0.10 (0.03)***	0.06 (0.03)*	−0.01 (0.03)
During the past 12 months, was there any time when you chose not to get the medical care you needed? (no vs. yes)	−0.07 (0.03)*	−0.04 (0.03)	0.04 (0.04)
I always tell my doctor the truth when they ask for information relevant to my healthcare (disagree vs. agree)	0.06 (0.06)	0.14 (0.05)**	−0.17 (0.08)*
Have you changed physicians in the past or sought a second opinion due to concerns about care? (no vs. yes)	−0.11 (0.03)***	0.00 (0.03)	0.05 (0.04)

Significance codes for p-values are as follows: <0.0001 = ***, ≥0.001 = **, <0.05 = *.

For each dependent variable, at least one subfactor from the TIMHSS was a statistically significant predictor, suggesting that the scale remains useful for explaining relevant attitudes and behaviors surrounding health care. Notably, the Doctor factor was the most consistently significant predictor of the criterion dependent variables. For instance, for each 1-unit increase in the Doctor scale (representing more distrust in doctors), the odds of not following doctor recommendations, refusing a new vaccine recommended by the doctor, choosing not to get necessary medical care, and changing physicians or asking for a second opinion were 1.1 times higher. The only exception was observed for always telling their doctor the truth, where the System and Policy factors were statistically significant but not the Doctor factor.

### 3.6 Measurement invariance – women vs. non-women

Within the sample, *n* = 254 respondents identified as women and *n* = 258 did not. Table 7, below, summarizes model fit indices for the configural, metric, and scalar invariant models.

**Table 7:** Comparison of original, metric, scalar, and residual invariant SEM three-factor models for women (*n* = 390) vs. non-women (350).

Model	DF	Chi-square scaled	RMSEA	CFI	TLI	SRMR	AIC	BIC
Three-factor	264	518.06	0.071	0.946	0.938	0.047	21,243.77	21,726.93
Full metric invariance	279	539.13	0.069	0.946	0.940	0.054	21,235.29	21,654.88
Full scalar invariance	294	561.06	0.068	0.945	0.943	0.054	21,222.96	21,578.98
Full residual invariance	312	582.27	0.067	0.944	0.945	0.055	21,222.09	21,501.82

Nested model comparisons using scaled chi-squared difference test with Satorra-Bentler estimation method. Robust test statistics reported for RMSEA, CFI, and SRMR.

The difference between the configural and metric invariant models was statistically insignificant (*p* = 0.19), as well as the metric and scalar invariant models (*p* = 0.21) and scalar and residual invariant models (*p* = 0.19). Between the models, differences were minimal for the robust CFI and RMSEA (−0.001), TLI (0.002), and SRMR (0.007, 0, and 0.001 respectively). Altogether, these results suggest that the 18-item TIMHSS has equal test validity between individuals who identify as women and those who do not.

## 4 Discussion

The present work responds to calls for health system leaders to “adopt evidence-based strategies to build the trusting relationships needed to address this complex social problem that robs people of their health and lives” (8) (p. 112). A key part of this evidence-base is a robust and practical trust scale. To develop and evaluate interventions, however, it is critical that we have measures that are valid and suit their intended purpose. The analysis in the present paper offers an 18-item measure of trust in doctors, health systems and health policy - the S-TIMHSS - that preserves the content, convergent, and criterion validity of the original 38-item version.

A measure of trust that reduces the burden on both the respondent and analyst is a valuable tool that can be used to inform interventions and evaluations to improve patient outcomes within healthcare settings. For example, many areas of healthcare use standardized surveys of patient satisfaction or experience, completed by patients, to provide feedback about the quality of care they receive [e.g., Canadian Institute for Health Information (37)]. These data are then used to support quality improvements and provide opportunity for (inter)national comparisons and benchmarking for the measure of patient experience. However, surveys of experience do not typically include measures of trust as an indicator of patient experience and this omission is problematic. Satisfaction can provide information on the care facility or interactions at the point of care. However, trust is a better predictor of behavior relevant to patient outcomes such as patient adherence and disclosure of information [e.g., see Birkhäuer, Gaab (7)]. The 18-item S-TIMHSS can be completed in under 3 min and can be used across health services – e.g., primary care services, public health units, or hospitals – to provide information that can be used to evaluate, build trust and ultimately shape health policy in future.

The S-TIMHSS is available upon request from the corresponding author. We recommend it for use in clinical settings as part of feedback reporting regarding patient experience, particularly when there is a desire to sum items within a factor to produce easily interpretable scores and to compare against other samples. Data collected using the measure can serve as a baseline for understanding patient trust, and in the ongoing evaluation of strategies implemented by providers and healthcare organizations to support the development of trust among patients. For example, data indicating that patient trust is impacted by perceived judgment on the part of their doctor, or concerns related to confidentiality, can be used to inform change at a provider and organization level. Data reflecting patient perceptions of the health system and health policy (i.e., waitlists, staffing/resources, rising costs) should be communicated to health system leaders to address structural determinants of trust that are amenable to change. The costs associated with a lack of patient trust might be a consideration in health economics modeling and used to guide policy. For example, health policy makers might consider the utility of trust-performance-indicators to gather evidence and investigate the cost-effectiveness of trust-building principles in healthcare organizations [see Gille, Maaß (38)]. Finally, the measure should also be used to investigate the role of trust in health behaviors of interest (e.g., medication adherence, vaccine uptake, service engagement) to identify where and how demonstrating trustworthiness of providers/services might be used in behavior change interventions.

### 4.1 Future directions in trust measurement

Within our work, measurement invariance could only be examined for gender identity, namely women versus non-women, as sample sizes were insufficient to permit testing of other groups. Future research will establish measurement invariance of the shortened TIMHSS for equity-deserving groups. In the evaluation of strategies to (re)build trust, it will be important that the measure is sensitive to change over time and thus establishing test–retest reliability of both the full and shortened versions of the TIMHSS is a potential direction for future research. There is also a need for prospective clinical studies to deepen our understanding of the complex interplay between trust and health outcomes (13). The S-TIMHSS can be used to generate data needed to further investigate this association. Lastly, it is beyond the scope of this paper to report methodologies for operationalizing data into strategies to build trust. However, readers may consult prior work combining measurement and community engagement approaches to the development and refinement of strategies to build trust [e.g., (39)]. The present work was conducted in Canada and as such, a potential limitation is that these questions may perform differently in other healthcare economies (e.g., in a predominately private system), countries of different income or infrastructure (low-and middle-income countries) or with different health beliefs and traditions. It is the hope of the authors that future studies evaluate the psychometric properties of adapted versions of the S-TIMHSS that reflect the system, context, and population of focus. We also acknowledge that there are factors for which we cannot control in the measurement of trust that should be considered in the performance of the S-TIMHSS in future research. For example, we cannot account for whether a respondent is speaking to trust in their own doctor (e.g., a family doctor with whom they meet regularly), specialists, or doctors in general (e.g., based on reputation). It is possible that the measure would behave differently if we were to examine trust after providing more specific details of the provider in question, or if the respondent was given a scenario upon which to base their response.

### 4.2 Concluding remarks

The S-TIMHSS provides a means for monitoring and being responsive to patient and public trust in doctors, health systems and health policy. However, on a more philosophical note, we acknowledge that this tool plays a small role in much larger social changes that need to occur to address what has been referred by some to as a trust crisis. This work comes several decades following a shift in society from a taken-for-granted trust in experts to an era where trust needs to be earned. The COVID−19 pandemic and spillover effects – e.g., a global economic crisis, growth in criticisms/distrust in science amidst disinformation and miseducation - have drastically changed what trust-building or trust-earning looks like. There is greater recognition, for example, that trust-building begins with ensuring the trustworthiness of healthcare providers and those developing and implementing health policies and system change (i.e., government representatives). As such, the health policy community has become refocused on trust as a matter of critical, real-world importance (13). We also need to continue to monitor the values underpinning public trust in policies and systems, as many of our questions might assume trust is predicated on health systems and policy working in the interest of the population as a whole, rather than an individual. For example, a trustworthy system for someone with financial means might be one that prioritizes services for those with the means to pay for them, instead of on the basis of need. These are the questions that we need to continue to ask/explore when measuring and responding to trust in different contexts. Beyond measurement, we need to continue to listen and engage with the public and their understandings of medicine and health systems.

## Data Availability

The datasets presented in this article are not readily available because sharing is not permitted in the ethics approval. Requests to access the datasets should be directed to samantha.meyer@uwaterloo.ca.
